# Two novel proteins, TtpB2 and TtpD2, are essential for iron transport in the TonB2 system of *Vibrio vulnificus*

**DOI:** 10.1002/mbo3.947

**Published:** 2019-10-08

**Authors:** Adel D. Barnes, Hailey J. Pfeifer, Brianne R. Zbylicki, Elena K. Roberts, Justin C. Rudd, Mario A. Manzo, Elysse A. Phillips, Michael M. Berry, Ryan J. Kenton

**Affiliations:** ^1^ Department of Biology University of Portland Portland OR USA

**Keywords:** iron, TonB, TtpB, TtpD, *Vibrio vulnificus*

## Abstract

In gram‐negative bacteria, energy‐dependent active transport of iron‐bound substrates across the outer membrane is achieved through the TonB systems of proteins. Three TonB systems have been identified in the human pathogen *Vibrio vulnificus*. The TonB1 system contains three proteins: TonB1, ExbB1, and ExbD1. Both the TonB2 and TonB3 systems have been shown to also contain a fourth protein, TtpC2 and TtpC3, respectively. Here, we report and begin to characterize two additional proteins in the TonB2 and TonB3 systems: TtpB and TtpD. Both TtpB2 and TtpD2 are absolutely required for the function of the TonB2 system in *V. vulnificus*. However, although both TtpB3 and TtpD3 in the TonB3 system are related to the proteins in the TonB2 system, neither are active in iron transport. All six protein components of the TonB2 system—TonB2, ExbB2, ExbD2, TtpB2, TtpC2, and TtpD2—are essential for the uptake of both endogenously produced iron‐bound siderophores and exogenous siderophores produced from other organisms. Through complementation, we have shown that *V. vulnificus* is capable of using different TtpD2 proteins from other *Vibrio* species to bring in multiple siderophores. In contrast, we also demonstrate that TtpB2 must come from *V. vulnificus,* and not other species within the genus, to complement mutations in the TonB2 system.

## Graphical abstract

We report and begin to characterize two additional proteins in *Vibrio vulnificus*' TonB2 and TonB3 systems: TtpB and TtpD. Both TtpB2 and TtpD2 are absolutely required for the function of the TonB2 system in *V. vulnificus* for growth in iron‐limited conditions as well as the uptake of both endogenous and exogenous siderophores. Through complementation, we have shown that *V. vulnificus* is capable of using different TtpD2 proteins from other *Vibrio* species; while in contrast, we also demonstrate that TtpB2 must come from *V. vulnificus*, and not other species within the genus, to complement mutations in the TonB2 system.

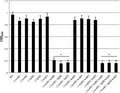

## 1 INTRODUCTION

*Vibrio vulnificus* is an opportunistic marine pathogen that can cause fatal septicemic disease in both humans and eels (Gulig, Bourdage, & Starks, 2005; Morris, 1988). Human infections are generally associated with eating contaminated seafood or through open wounds that are exposed to contaminated seawater (Linkous & Oliver, 1999; Strom & Paranjpye, 2000). Fatal primary septicemia can progress rapidly, resulting in a mortality rate of >50% within days. In humans, *V. vulnificus* preferentially infects those who have pre‐existing conditions associated with elevated iron levels, including cirrhosis, hemochromatosis, and thalassemia (Gulig et al., 2005).

*Vibrio vulnificus* strains are divided into three biotypes: 1, 2, and 3. Biotypes 1 and 3 are known as opportunistic pathogens in humans while the Biotype 2 is primarily an eel pathogen, and only particular isolates have been implicated in human infection (Amaro & Biosca, 1996; Bisharat et al., 1999; Efimov et al., 2013; Hor, Gao, & Wan, 1995; Tison, Nishibuchi, Greenwood, & Seidler, 1982). Although biochemically and serologically different, these three biotypes share common virulence features including iron‐regulated systems (Amaro, Biosca, Fouz, & Garay, 1992; Amaro, Biosca, Fouz, Toranzo, & Garay, 1994; Ran & Rhee, 2003).

Iron is an essential element required for survival by nearly every living organism. Iron can be highly versatile, acting as both an electron donor and an electron acceptor, as well as being involved in processes ranging from cell signaling to metabolism (Crosa, 1997). Although it is extremely abundant in the Earth's crust, iron remains very difficult for microorganisms to utilize. In aerobic conditions, iron readily forms insoluble ferric hydroxides (Crosa, Mey, & Payne, 2004). Further, microbes that colonize vertebrate animals must deal with the host's high‐affinity, iron‐binding proteins including transferrin, lactoferrin, and iron‐porphyrin complexes such as those found in hemoglobin (Bullen, Rogers, & Griffiths, 1978). In order to survive and cause infections within these hosts, microorganisms have evolved iron‐sequestering systems. These systems are centered around siderophores—small, high‐affinity iron‐chelating molecules—and the cell surface receptors for these siderophores (Actis, Tolmasky, & Crosa, 1999; Crosa, 1980; Köster, Actis, Waldbeser, Tolmasky, & Crosa, 1991).

Gram‐negative bacteria, such as *V. vulnificus*, possess an outer membrane (OM) that contains a large assortment of protein receptors. These receptors bind nutrients, minerals, and iron‐bound siderophore complexes. The inner membrane (IM) is where the energy for the cell is derived. This can be in the form of adenosine triphosphate (ATP) or the proton motive force (PMF), which provides the energy needed to power the uptake of iron‐bound siderophores. The periplasmic space between these two membranes presents a challenge to gram‐negative bacteria. In order to breach that space, energy created by the PMF is transduced to the OM through a group of proteins known as the TonB system (Braun, 1995; Crosa et al., 2004; Postle, 2007).

The TonB system has been extensively studied in *Escherichia coli*, where it has been shown to contain three integral IM proteins: TonB, ExbB, and ExbD (Bassford, Bradbeer, Kadner, & Schnaitman, 1976; Bradbeer & Woodrow, 1976; Braun et al., 1996). Besides providing stabilization, both ExbB and ExbD have been shown to transduce the PMF energy to TonB, thus causing a conformational change in TonB. This new conformation allows TonB to span the periplasmic space and make contact with TonB‐dependent OM receptors allowing the uptake of iron‐bound siderophores (Ahmer, Thomas, Larsen, & Postle, 1995; Germon, Ray, Vianney, & Lazzaroni, 2001; Higgs, Myers, & Postle, 1998; Kuehl & Crosa, 2009; Larsen et al., 2007; Larsen, Wood, & Postle, 1993; Ollis, Manning, Held, & Postle, 2009; Postle & Larsen, 2007; Swayne & Postle, 2011). Every member of the *Vibrionaceae* family contains two TonB systems: the TonB1 and TonB2 systems. The TonB1 system is very similar to *E. coli* in that it contains three proteins: ExbB, ExbD, and TonB. The TonB2 system has previously been shown to contain a fourth protein, TtpC2 (Kuehl & Crosa, 2009, 2010; Kustusch, Kuehl, & Crosa, 2011, 2012; Stork, Otto, & Crosa, 2007). When grown in iron‐limiting conditions, both the TonB1 and TonB2 systems have been shown to be up‐regulated, while iron‐rich conditions down‐regulate expression (Alice, Naka, & Crosa, 2008). In addition, some *Vibrio* species have been shown to contain a third TonB system. The TonB3 system looks very similar to the TonB2 system in that, along with the all of the proteins found in the TonB1 system, it also has previously been shown to contain a fourth protein, TtpC3 (Kuehl & Crosa, 2009, 2010; Kustusch, Kuehl, & Crosa, 2011, 2012). Unlike the TonB1 and TonB2 systems, the TonB3 system does not appear to be regulated by iron‐limiting conditions, but instead expression of this system has only been shown when the organism is grown in human serum (Alice et al., 2008).

We describe here the previously uncharacterized genes, *ttpB2* and *ttpD2*, in the TonB2 system of *V. vulnificus*. The gene *ttpB2* is located immediately upstream of *ttpC2* and is the first gene in the *tonB2* operon. In contrast, *ttpD2* is the last gene in the *tonB2* operon and is found downstream of *tonB2*. Homologs of both genes are found as part of *tonB2‐*like systems in other *Vibrio* species examined. Similar uncharacterized genes, *ttpB3* and *ttpD3*, have also been observed in the operon of the TonB3 system of *V. vulnificus*. Previous reports have indicated that both *ttpB2* and *ttpD2* are up‐regulated, along with the rest of the genes within the TonB2 system, in *V. vulnificus* during iron‐limiting conditions (Alice et al., 2008). Together, this suggests that both genes play vital roles in the TonB2*‐*mediated iron transport of *V. vulnificus.*

In this study, we demonstrate that both TtpB2 and TtpD2 are essential in the TonB2 system of *V. vulnificus.* We demonstrate that both proteins are necessary for growth in iron‐deplete conditions. In addition, we show that both TtpB2 and TtpD2 are essential for the uptake of both *V. vulnificus*‐produced endogenous siderophores and exogenous siderophores created by other microorganisms. We have determined that the TtpB3 and TtpD3 proteins from the TonB3 system cannot complement respective mutations in the TonB2 system. Finally, we show that the TtpD2 proteins from other *Vibrio* species can complement a Δ*ttpD2* mutation and function as a part of the TonB2 system of *V. vulnificus*, underlying the importance and ubiquity of this protein. In contrast, TtpB2 proteins from other *Vibrio* species cannot complement a *ttpB2* mutant.

## 2 MATERIALS AND METHODS

### 2.1 Bacterial strains, plasmids, and growth conditions

The bacterial strains and plasmids used in this study are listed in Table A1. *V. vulnificus* was routinely grown in tryptic soy broth with the addition of 10 g/liter of NaCl for a total of 1.5% NaCl (TSBS) or in minimal CM9 media (1 × M9 salts is 60 g Na_2_HPO_4_, 30 g KH_2_PO_4_, 50 g NaCl, 10 g NH_4_Cl per liter [pH 7.2], 0.2% casamino acids, 0.5% glucose, 10 μM CaCl_2_, 100 μM MgSO_4_; Crosa, 1980). *E. coli* was routinely grown in LB broth. Agar was added at 15 g/L when appropriate. Antibiotics were used at the following final concentrations: 30 μg/ml chloramphenicol (Cm) and 50 µg/ml kanamycin (Km^r^) for *E. coli*, and 10 μg/ml Cm for *V. vulnificus*, when appropriate. Thiosulfate‐citrate‐bile salts‐sucrose agar (TCBS; Becton, Dickinson) was used as the selective medium for *V. vulnificus* during conjugations. For iron‐limiting conditions, 2,2′‐dipyridyl was added at indicated concentrations. Iron‐rich conditions were obtained by the addition of ferric ammonium citrate (FAC) at indicated concentrations.

### 2.2 Sequence identity

The percent identity matrices and protein alignments were generated through Clustal Omega (Sievers et al., 2011). Protein sequences came from *V. vulnificus* CMCP6 (TtpB2 accession number WP_011081323, TtpD2 accession number AAO07317, TtpB3 accession number AAO09346, TtpD3 accession number WP_011078917), *V. vulnificus* biotype 2 ATCC 33149 (TtpB2 accession number WP_039446789, TtpD2 accession number WP_039446781), *V. cholerae* 01 Biovar El Tor (TtpB2 accession number NP_231188, TtpD2 accession number NP_231183), *V. parahaemolyticus* RIMD (TtpB2 accession number NP_799661, TtpD2 accession number NP_799666), *V. alginolyticus* 12G01 (TtpB2 accession number EAS74533, TtpD2 accession number EAS74528), and *V. anguillarum* 775 (TtpB2 accession number AEH33201, TtpD2 accession number AEH33206).

### 2.3 Construction and complementation of *V. vulnificus* mutants

In‐frame deletions of the entire coding sequences of genes were generated using splicing by overlap extension (SOE) PCR (Higuchi, Krummel, & Saiki, 1988; Senanayake & Brian, 1995). Upstream and downstream regions (approximately 700 bp) flanking each gene were amplified with specific primers (Table A2, Primers 1/3 and 2/4). The two 700‐bp fragments with overlapping primer ends were spliced using SOE PCR with the two outside primers (Primers 1/2). The amplified fragment was cloned into the pCR2.1 vector (Invitrogen), digested with appropriate restriction enzymes, and subcloned into the suicide vector pDM4, which had been previously digested with the same restriction enzymes. *E. coli* S17‐1 λ*pir* transformed with the pDM4 (Milton, O'Toole, Horstedt, & Wolf‐Watz, 1996) derivatives was conjugated with *V. vulnificus*, and exconjugants were selected on TCBS agar with 2 μg/ml Cm. For the excision of the suicide vector, clones were incubated in the absence of Cm and plated on TSAS plates (TSBS with 1.5% agar) containing 15% sucrose. Colonies that grew on these plates were screened for Cm sensitivity, and deletions within the genes of interest were confirmed by PCR.

Complementation of deleted genes was achieved by amplifying each gene of interest by PCR with primers containing restriction sites (Table A2). The fragments were cloned into pCR2.1 vector (Invitrogen), sequenced, and then subcloned into pMMB208 (Morales, Bäckman, & Bagdasarian, 1991) under the control of the *Ptac* promoter. This promoter is under the control of the *lacI* gene harbored in the vector. The constructs were transformed into *V. vulnificus* strains by triparental conjugation with the plasmid helper pRK2013 (Figurski & Helinski, 1979). In order to induce transcription of the cloned genes, 1 mM isopropyl‐d‐thiogalactopyranoside (IPTG) was added to the solid and/or broth medium.

### 2.4 Growth assays

Overnight cultures were diluted to an optical density at 600 nm (OD_600_) of 0.02 into 25 ml of either TSBS or CM9 media and incubated at 37°C while shaking at 160 rpm. Readings were taken every 30 min for 300 min. For iron‐limiting conditions, 75 µM 2,2′‐dipyridyl was added to CM9 media. Complementation growth assays also contained 10 µg/ml chloramphenicol and 1 mM IPTG.

### 2.5 CAS assays

Overnight cultures were diluted to an optical density at 600 nm (OD_600_) of 0.02 into 25 ml of CM9 media and incubated at 37°C to an OD_600_ of 0.5 (∼3 hr). Cells were normalized to an OD_600_ of 0.5 in 1 ml of CM9 media. Cells were then centrifuged, and 0.5 ml of supernatant was used in the chrome azurol S (CAS) assay. A ratio of 1:1 of cell supernatant to CAS solution (600 μM hexadecyltrimethylammonium bromide [HDTMA], 150 μM FeCl_3_, 150 μM CAS) was mixed and placed into 0.2‐cm cuvettes, and the OD_630_ was read using a spectrophotometer at 20 min after mixing. The assay was normalized to the wild type (WT), and the final values were inverted to show a positive change for ease of reading.

### 2.6 Bioassays

Bacteria were grown overnight in TSBS with the appropriate antibiotics and diluted 1/500 into TSAS containing 100 µM 2′2‐dipyridyl. Complemented mutants also had appropriate antibiotics added as well as 1 mM IPTG. In these experiments, bacterial cells were included in the agar and, upon solidification, plates were spot inoculated with different iron sources. After incubation of the plates at 37°C for 24 hr, halos of bacterial growth surrounding the locations of the spots indicated positive results. In these experiments, ferric ammonium citrate (FAC), which does not require active transport to obtain, was included to confirm that the strain inoculated in the agar was viable. The purified compounds spotted on top of the bioassay plates to determine the functionality of the different TonB cluster of genes in *V. vulnificus* were as follows: 1 mg/ml iron‐free vibriobactin (EMC Microcollections), 1 mg/ml iron‐free aerobactin, 1 mg/ml iron‐free enterobactin, 1 mg/ml iron‐free ferrioxamine (all three from EMC Microcollections), and 500 μg/ml FAC (Sigma). Each bioassay plate was spotted with 2 μl of each indicated iron source. To test vibrioferrin, WT *V. parahaemolyticus* was streaked onto the plate. Vulnibactin and the hydroxamate siderophore, both from *V. vulnificus*, were tested by streaking either WT or the Δ*venB* strain onto the bioassay plate. Halo growth around these streaks indicates that the strain within the plate can use the siderophore being produced by the streaked strain.

### 2.7 RNA extractions

Wild‐type *V. vulnificus* CMCP6 was grown overnight in TSBS and diluted 1/400 in CM9 with the addition of 250 µg/ml FAC for iron‐rich conditions or with the addition of 50 µM 2′2‐dipyridyl for iron‐limited conditions. Cultures were grown to an optical density at 600 nm (OD_600_) of 0.3. Samples were centrifuged, and RNA was harvested using Qiagen's RNeasy kit with Qiagen's RNAprotect following manufacturer's instructions. TurboDNase (Thermofisher) was used, and manufacturer's protocols were followed to ensure complete degradation of genomic DNA. At least three biological replicates were created for each condition.

### 2.8 Semi‐quantitative RT‐PCR

Purified RNA samples underwent reverse transcription to synthesize cDNA using the Qiagen QuantiTect Reverse Transcription kit following manufacturer's instructions. Control reactions without reverse transcriptase were also performed for each sample. Reverse transcriptase PCRs (RT‐PCRs) were then performed using 2 μl of each reverse transcription (RT) reaction mix using gene‐specific primers described in Table A2. A control without reverse transcriptase enzyme in the RT reaction mix was used for each PCR. Fragments were resolved by electrophoresis on agarose gels. At least three replicates from each biological replicate were created. Pictures of gels were taken under UV light with a Kodak Gel Logic 2200 imaging system at an exposure time of 0.1 s. Images were then analyzed using ImageJ software. Numerical values for each gene could then be normalized to our control gene glyceraldehyde 3‐phosphate dehydrogenase (GAPDH) for each band.

## 3 RESULTS

### 3.1 *vulnificus* contains two novel proteins associated with the TonB2 and TonB3 systems

**Figure 1:**
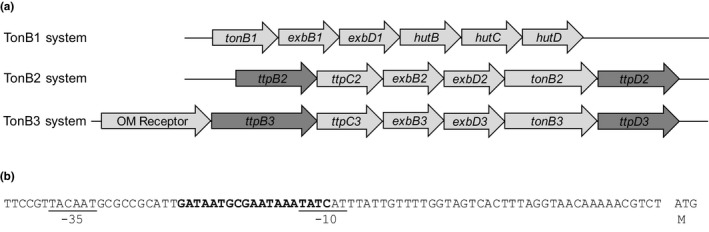
The three TonB systems of *V. vulnificus* and the promoter region of the TonB2 system. (a) The TonB1, TonB2, and TonB3 systems of *V. vulnificus* are depicted with the novel genes, *ttpB* and *ttpD*, noted in dark gray. (b) Nucleotide sequence analysis in the intergenic region immediately upstream of the TonB2 operon shows the putative Fur box sequence (bold) and the predicted −10 and −35 regions (underlined). The translational start site of *ttpB2* is indicated by the ATG codon and the methionine (M) designation

The bacterium *V. vulnificus* contains three TonB systems spread across its two chromosomes (Figure 1a; Kustusch et al., 2011; Tagomori, Lida, & Honda, 2002). The TonB1 system, present in all members of the *Vibrionaceae*, consists of three genes: *tonB1, exbB1,* and *exbD1*. This system is always found associated with the genes needed for heme uptake—*hutB, hutC,* and *hutD* (Figure 1a; Henderson & Payne, 1994; Kuehl & Crosa, 2010). The TonB1 system found in *Vibrio* is very similar to the well‐studied TonB system of *E. coli*.

All bacteria in the *Vibrio* genus examined to date also contain a second TonB system, the TonB2 system. This second system has shown uptake specificity to certain iron‐bound substrates in *V. vulnificus,* as well as *V. cholerae* and *V. anguillarum* (Kuehl & Crosa, 2010; Kustusch et al., 2012; Stork et al., 2007). Three proteins similar to those found in the TonB1 system—TonB2, ExbB2, and ExbD2—are found in this system. In addition, a fourth protein, TtpC2, has been shown to be essential for the function of the TonB2 system in multiple *Vibrio* species (Kuehl & Crosa, 2009; Kustusch et al., 2012; Stork et al., 2007). It has been alluded to, but never shown, that two additional genes may be a part of the TonB2 system (Kustusch et al., 2012). DNA sequence analysis of the *tonB2* operon, located on *V. vulnificus*' second, larger chromosome, suggested that two open reading frames (ORFs) are present as the first and last ORFs in the *tonB2* operon. Each appears to encode for hypothetical proteins of unknown function. We have designated the first ORF in this operon as *ttpB2,* and the last ORF as *ttpD2* for, TonB2 complex‐associated transport protein B and D (Figure 1a).

The *tonB2* operon therefore appears to contain six genes, beginning with *ttpB2* (Figure 1a). A detailed analysis of the nucleotide region immediately upstream of *ttpB2* is shown in Figure 1b. A potential ferric uptake regulator (Fur) binding site (shown in bold) and promoter region (underlined) are clearly identified before the translational start site of TtpB2. The *fur* sequence identified is 78.95% similar to the *fur* consensus sequence 5′‐gataatgataatcattatc‐3′ (Lavrrar & McIntosh, 2003). Fur is a DNA‐binding protein that recognizes and binds Fur binding sites. Together with Fe^2+^, as a corepressor, this protein complex can block transcription of target genes (Troxell & Hassan, 2013). These results suggest that *ttpB2* is the first gene in the *tonB2* operon and suggests that the lack of iron would induce transcription of these genes.

**Figure 2:**
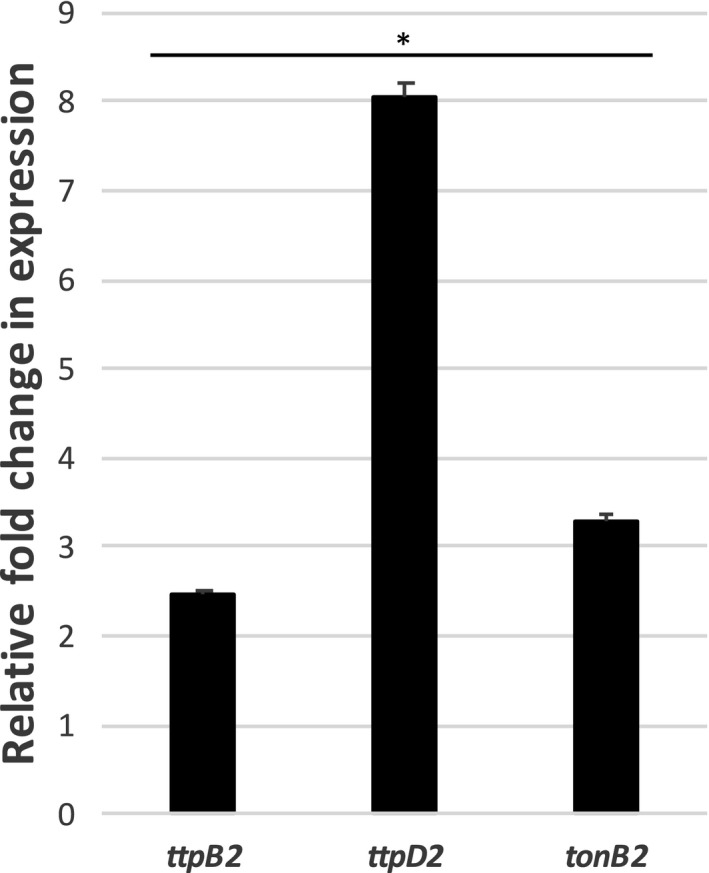
*ttpB2* and *ttpD2* are transcriptionally activated in iron‐deplete conditions along with *tonB2*. Relative fold changes in expression of *ttpB2, ttpD2,* and *tonB2* from the TonB2 operon are shown. Semi‐quantitative reverse transcriptase PCR (RT‐PCR) was performed in triplicate on at least three biological replicates. Gel images were analyzed by ImageJ software to determine total pixels in each band. Fold changes in expression are the average values of iron‐limiting conditions compared to iron‐rich conditions normalized to glyceraldehyde 3‐phosphate dehydrogenase (GAPDH). Significance (*p < .005) was determined through Student's *t* test

To determine whether the TonB2 system, specifically the *ttpB2* and *ttpD2* genes, is transcribed at elevated levels during iron‐limiting conditions, semi‐quantitative reverse transcriptase (RT) PCR was performed. We saw a ~2.5‐fold and ~8‐fold elevated expression of *ttpB2* and *ttpD2*, respectively, in iron‐depleted conditions compared to iron‐rich conditions (Figure 2). We used *tonB2* expression (~3‐fold increase) as a positive control. The housekeeping gene glyceraldehyde 3‐phosphate dehydrogenase (GAPDH) was used for normalization. These results are very similar to what has been seen in another study (*ttpB2 *= *~*5‐fold, *ttpD2 *= ~8‐fold, and *tonB2 *= ~4‐fold increase) using microarray analysis (Alice et al., 2008). Together, these results clearly show that both *ttpB2* and *ttpD2* are induced by iron‐depleted conditions and are a part of the TonB2 system.

**Figure A1:**
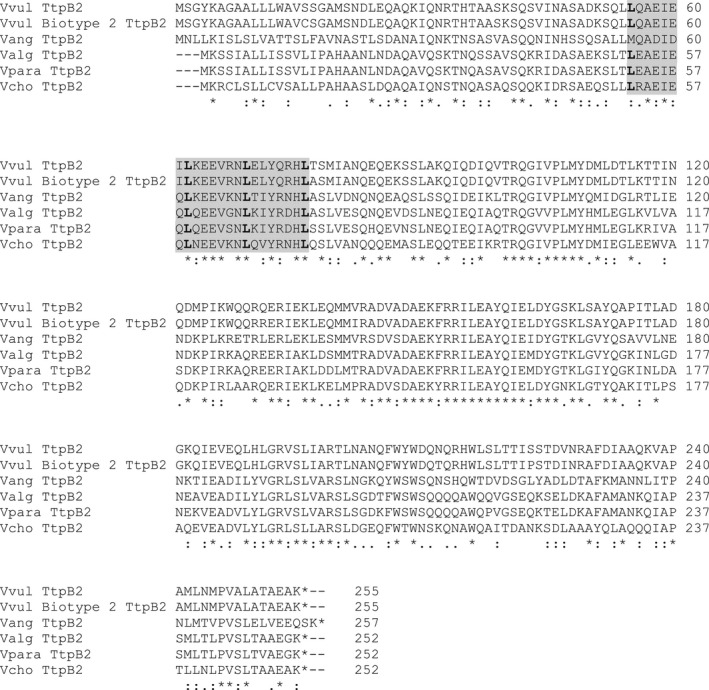
Sequence alignments of TtpB2 and their homologs. A ClustalO (Sievers et al., 2011) sequence alignment was performed using TtpB2 from: *V. vulnificus* CMCP6 (*Vvul* TtpB2); *V. vulnificus* VSSD100 Biotype 2 (*Vvul* Biotype 2 TtpB2); *V. anguillarum* 775 (*Vang* TtpB2); *V. alginolyticus* 12G01 (*Valg* TtpB2); *V. parahaemolyticus* RIMD (*Vpara* TtpB2); and *V. cholerae* CA401 (*Vcho* TtpB2). Numbering refers to the amino acid position in the primary sequence of the respective proteins. Conserved residues are marked with an asterisk (*), similar residues are annotated by a period (.), and a colon (:) indicates conservation between groups of strongly similar properties. The leucine zipper motif is highlighted in gray with the leucine (L) residues bolded

The *ttpB2* gene encodes for a hypothetical protein with a large domain of unknown function (DUF3450). This family of proteins, DUF3450, appears in both eukaryotes and bacteria and always contains about 260 amino acids (Altschul, Gish, Miller, Myers, & Lipman, 1990). This is very similar to the length of *ttpB2*, which encodes for 255 amino acids in *V. vulnificus*. The gene, *ttpB2*, also appears to be the first ORF in all members of the *Vibrionaceae's* TonB2 systems examined. A protein alignment (Figure A1) as well as a percent identity matrix (Table 1) compares *V. vulnificus* to four other *Vibrio* species as well as a Biotype 2 isolate of *V. vulnificus*. The *V. vulnificus* strain examined in this study is a Biotype 1 isolate, and its TtpB2 protein is extremely similar to the Biotype 2's TtpB2 (97.3%). When compared to other *Vibrio* species, the average similarity was about 50% (Table 1). This similarity is comparable to the rest of the known proteins in the TonB2 system (TonB2 ~ 45%, ExbB2 ~ 63%, ExbD ~ 86%, TtpC2 ~ 59%) when one compares *V. vulnificus* to these same four species (Kustusch et al., 2012). Finally, a leucine zipper motif can be found near the N‐terminus in all of these homologs (Figure A1; Altschul et al., 1990). Leucine zippers are used for protein–protein interactions and are located in alpha helices where the pattern Leu‐X6‐Leu‐X6‐Leu‐X6‐Leu is found (Hirst, Vieth, Skolnick, & Brooks, 1996).

**Table 1:** Percent identity matrix of TtpB2 using different *Vibrio* species

TtpB2	*V. vul* (%)	*V. vul* Biotype 2 (%)	*V. cho* (%)	*V. ang* (%)	*V. alg* (%)	*V. para* (%)	*V. vul* TonB3 System (%)
*V. vul*	100						
*V. vul* Biotype 2	97.3	100					
*V. cho*	52.4	52.0	100				
*V. ang*	46.7	46.3	53.2	100			
*V. alg*	50.0	50.4	62.7	55.6	100		
*V. para*	50.4	50.4	62.7	52.4	92.1	100	
*V. vul* TonB3 system	30.3	29.9	33.9	27.5	33.5	34.3	100

Note

Percent identity matrices generated using ClustalO (Sievers et al., 2011) are shown using TtpB2. Protein sequences of TtpB2 were compared against: *V. vulnificus* CMCP6 (*V. vul*); *V. vulnificus* VSSD100 Biotype 2 (*V. vul* Biotype 2); *V. cholerae* CA401 (*V. cho*); *V. anguillarum* 775 (*V. ang*); *V. alginolyticus* 12G01 (*V. alg*); and *V. parahaemolyticus* RIMD (*V. para*). The TtpB3 protein from *V. vulnificus* CMCP6 (*V. vul* TonB3 System) is also compared.

**Figure A2:**
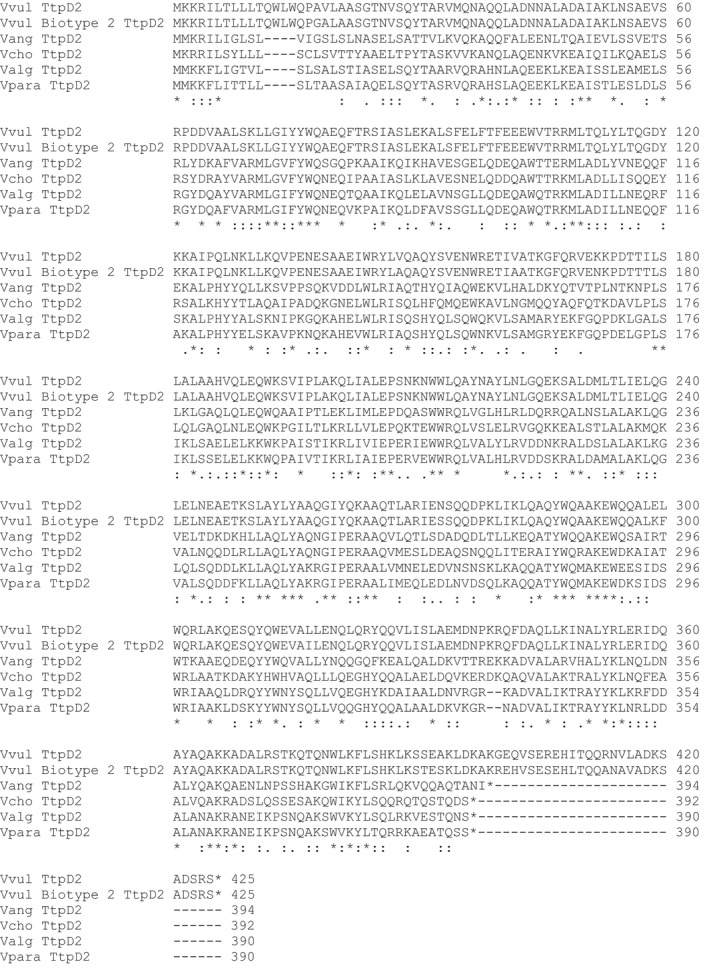
Sequence alignments of TtpD2 and their homologs. A ClustalO (Sievers et al., 2011) sequence alignment was performed using TtpD2 from: *V. vulnificus* CMCP6 (*Vvul* TtpD2); *V. vulnificus* VSSD100 Biotype 2 (*Vvul* Biotype 2 TtpD2); *V. anguillarum* 775 (*Vang* TtpD2); *V. cholerae* CA401 (*Vcho* TtpD2); *V. alginolyticus* 12G01 (*Valg* TtpD2); and *V. parahaemolyticus* RIMD (*Vpara* TtpD2). Numbering refers to the amino acid position in the primary sequence of the respective proteins. Conserved residues are marked with an asterisk (*), similar residues are annotated by a period (.), and a colon (:) indicates conservation between groups of strongly similar properties

The *ttpD2* gene encodes for a hypothetical 425 amino acid protein. It contains a number of potential tetracopeptide repeat (TPR) motifs throughout the protein (Altschul et al., 1990). TPR motifs are structural motifs present in a wide range of proteins. They generally mediate protein–protein interactions and help assemble larger multiprotein complexes (Schultz, Milpetz, Bork, & Ponting, 1998). As with *ttpB2*, *ttpD2* also appears in all members of the *Vibrionaceae* examined. A protein alignment (Figure A2) and a percent identity matrix (Table 2) compare *V. vulnificus* to four other *Vibrio* species as well as a Biotype 2 isolate of *V. vulnificus*. Overall, TtpD2 had a much lower similarity (35%) to other *Vibrio* species as compared to TtpB2 or any of the other four proteins in the TonB2 system (Table 2; Kustusch et al., 2012). As with TtpB2, TtpD2 also had a very high similarity (95.5%) to the TtpD2 protein in the *V. vulnificus* Biotype 2 strain (Table 2). Unlike TtpB2, where large stretches of similarity were observed (Figure A1), TtpD2 had only very short stretches of homology between the different TtpD2 proteins found in the other four *Vibrio* species (Figure A2).

**Table 2:** Percent identity matrix of TtpD2 using different *Vibrio* species

TtpD2	*V. vul *(%)	*V. vul* Biotype 2 (%)	*V. cho *(%)	*V. ang *(%)	*V. alg *(%)	*V. para *(%)	*V. vul* TonB3 system (%)
*V. vul*	100						
*V. vul* Biotype 2	95.5	100					
*V. cho*	34.7	34.7	100				
*V. ang*	37.3	37.3	52.0	100			
*V. alg*	34.1	34.4	55.4	53.1	100		
*V. para*	34.9	35.4	54.6	53.3	82.1	100	
*V. vul* TonB3 system	23.0	23.5	24.3	24.8	22.9	23.4	100

Note

Percent identity matrices generated using ClustalO (Sievers et al., 2011) are shown using TtpD2. Protein sequences of TtpD2 were compared against: *V. vulnificus* CMCP6 (*V. vul*); *V. vulnificus* VSSD100 Biotype 2 (*V. vul* Biotype 2); *V. cholerae* CA401 (*V. cho*); *V. anguillarum* 775 (*V. ang*); *V. alginolyticus* 12G01 (*V. alg*); and *V. parahaemolyticus* RIMD (*V. para*). The TtpD3 protein from *V. vulnificus* CMCP6 (*V. vul* TonB3 System) is also compared.

A third TonB3 system has been identified in *V. vulnificus* as well as in a number of *Vibrio* species and species within the genera *Alivibrio, Photobacterium,* and *Teredinibacter* (Kustusch et al., 2011). This system appears to contain the six proteins found in the TonB2 system (Figure 1a). In contrast to the TonB2 system, a gene encoding an outer membrane (OM) receptor protein can be found at the beginning of the *tonB3* operon. TtpB2 and TtpD2 were compared to their counterparts in the TonB3 system. Only a 30.3% similarity was found between the TtpB2 and TtpB3 proteins and a 23% similarity between TtpD2 and TtpD3 (Tables 1 and 2). This is very similar to homologies in other known TonB2 system proteins when comparing *V. vulnificus*' TonB2 system against its TonB3 system: TonB 27%, ExbB 40%, ExbD 57%, and TtpC 37% (Kustusch et al., 2012).

### 3.2 Both TtpB2 and TtpD2 are necessary components of the TonB2 system in mediating growth and the transport of endogenous siderophores

**Figure 3:**
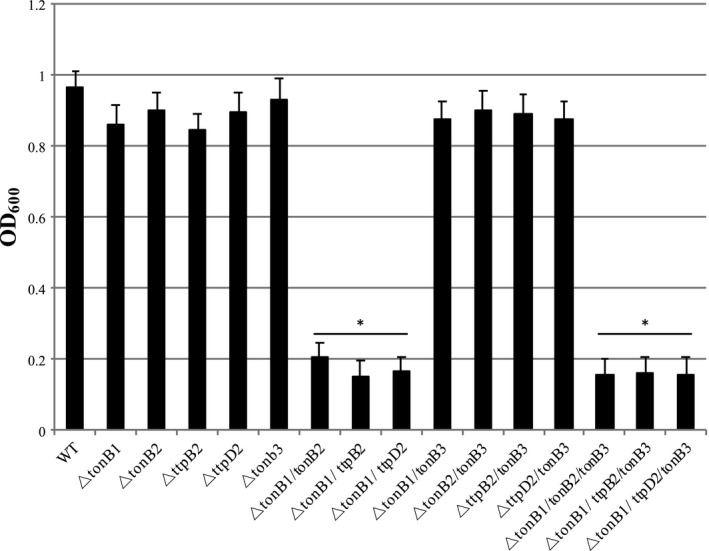
TtpB2 and TtpD2 are necessary components of the TonB2 system for growth in iron‐limited conditions. Overnight cultures were diluted to an optical density at 600 nm (600_nm_) of 0.02 into CM9 media plus 75 µM 2,2′‐dipyridyl (iron‐deplete media). Cultures were grown at 37°C and monitored for growth every 30 min. The average growth at 150 min is depicted for each strain. Strains were grown in triplicate, and significance (*p = <.005) was determined through Student's *t* test

Growth assays were used to determine the necessity and essentiality of TtpB2 and TtpD2 in the TonB2 system. An assortment of mutants was grown under iron‐rich and iron‐deplete conditions. When grown in iron‐rich media, TSBS, or minimal media, CM9, no differences in growth could be seen (data not shown). In contrast, when grown in CM9 plus the iron chelator dipyridyl (iron‐deplete conditions), a considerable difference in growth was noted in strains missing components of both the TonB1 and TonB2 systems (Figure 3). Single mutants in any of the TonB systems grew as well as wild‐type. Double mutants, Δ*tonB1* Δ*tonB2,* Δ*tonB1* Δ*ttpB2*, and Δ*tonB1* Δ*ttpD2*, showed a considerable growth deficit, as did the triple mutants Δ*tonB1* Δ*tonB2* Δ*tonB3*, Δ*tonB1* Δ*ttpB2* Δ*tonB3,* and Δ*tonB1* Δ*ttpD2* Δ*tonB3*. In all of these strains, both the TonB1 and TonB2 systems were missing components of their respective system. The double and triple mutant strains deleted for *ttpB2* or *ttpD2* showed the same lack of growth as strains deleted for *tonB2*. In addition, any double mutant strain that left either the TonB1 or the TonB2 system functional (Δ*tonB1* Δ*tonB3,* Δ*tonB2* Δ*tonB3*, Δ*ttpB2* Δ*tonB3,* and Δ*ttpD2* Δ*tonB3*) retained the ability to grow as well as the wild‐type strain and single mutants. This shows the importance and redundancy of the TonB1 and TonB2 systems when grown in these conditions and the inability of the TonB3 system to be used to overcome the loss of either of these systems. These data demonstrate the essentiality of TtpB2 and TtpD2 in the TonB2 system for growth in iron‐depleted conditions.

Bioassays were used to investigate the essentiality of TtpB2 and TtpD2 within the TonB2 system for the uptake of endogenous siderophores. *V. vulnificus* can produce two siderophores, vulnibactin, and the uncharacterized hydroxamate siderophore (Simpson & Oliver, 1983). An assortment of mutants was tested with various combinations of deletions in different TonB systems (Table 3). We have previously tested the role of TtpC2 in the TonB2 system, but not TtpB2 nor TtpD2 (Kustusch et al., 2012).

**Table 3:** TtpB2 and TtpD2 are essential components of the TonB2 system mediating the uptake of endogenous siderophores in *V. vulnificus*

*V. vulnificus* strain or genotype	Growth on indicated iron sources^a^		
	FAC	Vulnibactin	Hydroxamate
WT	+	+	+
Δ*tonB1*	+	+	+
Δ*tonB2*	+	+	+
Δ*ttpB2*	+	+	+
Δ*ttpD2*	+	+	+
Δ*tonB3*	+	+	+
Δ*tonB1* Δ*tonB2*	+	−	−
Δ*tonB1* Δ*ttpB2*	+	−	−
Δ*tonB1* Δ*ttpD2*	+	−	−
Δ*tonB1* Δ*tonB3*	+	+	+
Δ*tonB2* Δ*tonB3*	+	+	+
Δ*ttpB2* Δ*tonB3*	+	+	+
Δ*ttpD2* Δ*tonB3*	+	+	+
Δ*tonB1* Δ*tonB2* Δ*tonB3* b	+	−	−
Δ*tonB1* Δ*ttpB2* Δ*tonB3*	+	−	−
Δ*tonB1* Δ*ttpD2* Δ*tonB3*	+	−	−
Δ*tonB1* Δ*ttpB2* Δ*tonB3* w/pMMB208	+	−	−
Δ*tonB1* Δ*ttpD2* Δ*tonB3* w/pMMB208	+	−	−
Δ*tonB1* Δ*ttpB2* Δ*tonB3* w/p*tonB1*	+	+	+
Δ*tonB1* Δ*ttpD2* Δ*tonB3* w/p*tonB1*	+	+	+
Δ*tonB1* Δ*ttpB2* Δ*tonB3* w/p*ttB2*(Vvul)	+	+	+
Δ*tonB1* Δ*ttpD2* Δ*tonB3* w/p*ttD2*(Vvul)	+	+	+
Δ*tonB1* Δ*ttpB2* Δ*tonB3* w/p*tonB3*	+	−	−
Δ*tonB1* Δ*ttpD2* Δ*tonB3* w/p*tonB3*	+	−	−

a Growth was determined by the presence of halos (+) or lack thereof (−) around each iron source indicated after 18 hr at 37°C. FAC was added as a positive control, as it does not require active transport and confirmed our imbedded strain was viable in each plate. Two microliters of FAC was spotted (500 µg/ml). Wild‐type *V. vulnificus* was streaked on the plate as a source of vulnibactin (the dominant siderophores produced), and the halo of growth was monitored around the streak. The *V. vulnificus* Δ*venB* mutant strain is deficient in the production of vulnibactin and was streaked onto the plate to access the use of the hydroxamate siderophore by each strain.

b The triple mutant strain Δ*tonB1* Δ*tonB2* Δ*tonB3* contained an additional mutation in the *lacZ* gene (Strain AA‐12 in Table A1). This mutation was necessary for previous studies and has been shown to not affect Fe‐siderophore transport (Alice et al., 2008).

It was previously shown that both the TonB1 and TonB2 systems, but not the TonB3 system, could transport both of the endogenous siderophores of *V. vulnificus* (Kustusch et al., 2012)*.* As shown in Table 3, WT, as well as any single mutant, could transport both native siderophores. Similar to the trend in the growth assay, any double mutant tested that left both the TonB1 and TonB2 systems missing a component of their respective system could not uptake endogenous siderophores. This was true when *tonB2*, *ttpB2*, or *ttpD2* was deleted in conjunction with a Δ*tonB1* mutant (Table 3). These results indicate that both TtpB2 and TtpD2 are necessary components of the TonB2 system when transporting endogenous siderophores. In addition, these data demonstrate that TtpB3 and TtpD3 cannot be used in place of TtpB2 or TtpD2, respectively, because both TtpB3 and TtpD3 were still available in these strains. Both of these siderophores were also unable to be used when all three TonB systems were deleted using a Δ*tonB1* Δ*tonB2* Δ*tonB3* strain, Δ*tonB1* Δ*ttpB2* Δ*tonB3* strain, or a Δ*tonB1* Δ*ttpD2* Δ*tonB3* strain (Table 3). To determine that the deletions did not have any polar effects and that the mutations were truly in the targeted genes, assorted complemented strains were also utilized in this assay. Endogenous siderophores could once again be brought into the cell when either the TonB1 system was complemented (w/p*tonB1*), or the TonB2 system was complemented (w/p*ttpB2* in a *ttpB2* deletion or w/p*ttpD2* in a *ttpD2* deletion), further confirming the importance of these proteins to the uptake of endogenous siderophores and the ability to use either (Table 3).

We further analyzed these mutants through the use of chrome azurol S (CAS) assays. Briefly, these colorimetric assays can be used to detect secreted iron‐bound complexes, like siderophores. When in iron‐depleted conditions, siderophores are secreted from *V. vulnificus* into the surrounding media. If the strain has a functional TonB system that can mediate the uptake of these now iron‐bound siderophores, they will be brought back into the cell. If the TonB systems are nonfunctional and the iron‐bound siderophores cannot be brought back into the cell, an accumulation builds up in the medium. By harvesting the supernatant of different TonB mutants and mixing it with CAS reagents, one can determine which strain is capable of siderophore uptake through a color change.

**Figure 4:**
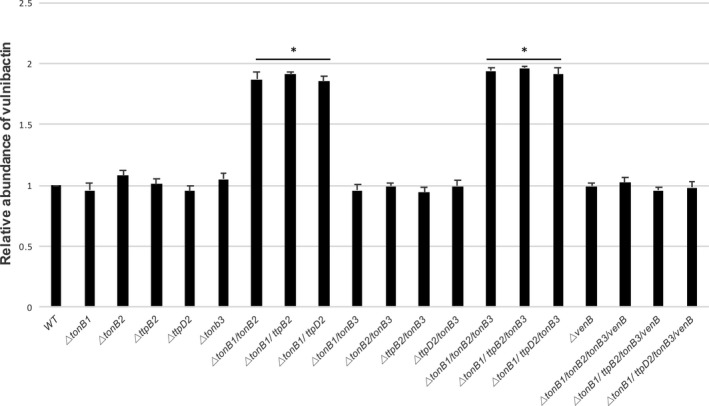
TtpB2 and TtpD2 within the TonB2 system are essential for vulnibactin uptake. Liquid chrome azurol S (CAS) assays were used to determine the relative production of vulnibactin, one of *V. vulnificus*' native siderophores. Supernatant from log phase cultures grown in CM9 minimal media was mixed in a 1:1 ratio with CAS solution. OD_630_ was determined after 20 min of incubation at room temperature and normalized to WT. Strains were grown in triplicate, and significance (*p = <.005) was determined through Student's *t* test

The results from these experiments can be seen in Figure 4. Confirming our bioassay results, both the TonB1 and TonB2 systems are used for the uptake of endogenous siderophores. Both single and double mutations that still allow either the TonB1 or the TonB2 system to function show the same amount of siderophore production as the wild‐type strain. Only when genes in both the TonB1 and TonB2 systems are deleted do we see an increase in relative production of endogenous siderophores. We see the same results in the double mutants when *tonB1* is deleted along with either *tonB2*, *ttpB2*, or *ttpD2*. This important result confirms the essentiality of both TtpB2 and TtpD2 in the TonB2 system.

It has previously been shown that vulnibactin, produced through an assortment of genes including *venB*, is the primary siderophore secreted under iron‐deplete conditions (Kustusch et al., 2012). In Figure 4, we see approximately the same levels of vulnibactin in the wild‐type strain compared to the Δ*venB* strain and the triple mutants in conjunction with Δ*venB*. This is due to the wild‐type strain bringing vulnibactin into the cell, leaving very little in the supernatant, and the mutant strain never producing vulnibactin to begin with. These results further show that the iron‐binding molecule that was both present in the supernatant of the double and triple mutants and caused an increase in values was indeed vulnibactin and not an artifact or the hydroxamate‐type siderophore.

### 3.3 Both TtpB2 and TtpD2 are essential for the TonB2 system in mediating the transport of exogenous siderophores

*Vibrio vulnificus* can thrive in a variety of environments and hosts, because of the evolved ability to use a diverse assortment of iron sources. These include the use of exogenous siderophores, that is, siderophores that are produced by other bacterial and fungal species (Alice et al., 2008; Aso, Miyoshi, Nakao, Okamoto, & Yamamoto, 2002). In order to evaluate any potential roles for TtpB2 and TtpD2 in the uptake of these exogenous siderophores by *V. vulnificus*, we performed bioassays with an assortment of mutants and complemented strains. Table 4 shows which exogenous siderophores can be utilized by assorted *V. vulnificus* mutants, and more specifically what effect *ttpB2* and *ttpD2* mutations has on the TonB2 system.

**Table 4:** TtpB2 and TtpD2 are essential components of the TonB2 system mediating the uptake of exogenous siderophores in *V. vulnificus*

*V. vulnificus* strain or genotype	Growth on indicated iron sources^a^					
	FAC	Vibriobactin	Vibrioferrin	Ferrioxamine	Enterobactin	Aerobactin
WT	+	+	+	+	+	+
Δ*tonB1*	+	+	+	+	+	+
Δ*tonB2*	+	+	+	+	+	−
Δ*ttpB2*	+	+	+	+	+	−
Δ*ttpD2*	+	+	+	+	+	−
Δ*tonB3*	+	+	+	+	+	+
Δ*tonB1* Δ*tonB2*	+	−	−	−	−	−
Δ*tonB1* Δ*ttpB2*	+	−	−	−	−	−
Δ*tonB1* Δ*ttpD2*	+	−	−	−	−	−
Δ*tonB1* Δ*tonB3*	+	+	+	+	+	+
Δ*tonB2* Δ*tonB3*	+	+	+	+	+	−
Δ*ttpB2* Δ*tonB3*	+	+	+	+	+	−
Δ*ttpD2* Δ*tonB3*	+	+	+	+	+	−
Δ*tonB1* Δ*tonB2* Δ*tonB3* b	+	−	−	−	−	−
Δ*tonB1* Δ*ttpB2* Δ*tonB3*	+	−	−	−	−	−
Δ*tonB1* Δ*ttpD2* Δ*tonB3*	+	−	−	−	−	−
Δ*tonB1* Δ*tonB2* Δ*tonB3* Δ*venB*	+	−	−	−	−	−
Δ*tonB1* Δ*ttpB2* Δ*tonB3* Δ*venB*	+	−	−	−	−	−
Δ*tonB1* Δ*ttpD2* Δ*tonB3* Δ*venB*	+	−	−	−	−	−
Δ*tonB1* Δ*ttpB2* Δ*tonB3* Δ*venB* w/pMMB208	+	−	−	−	−	−
Δ*tonB1* Δ*ttpD2* Δ*tonB3* Δ*venB* w/pMMB208	+	−	−	−	−	−
Δ*tonB1* Δ*ttpB2* Δ*tonB3* Δ*venB* w/p*tonB1*	+	+	+	+	+	−
Δ*tonB1* Δ*ttpD2* Δ*tonB3* Δ*venB* w/p*tonB1*	+	+	+	+	+	−
Δ*tonB1* Δ*ttpB2* Δ*tonB3* Δ*venB* w/p*ttpB2*(Vvul)	+	+	+	+	+	+
Δ*tonB1* Δ*ttpD2* Δ*tonB3* Δ*venB* w/p*ttpD2*(Vvul)	+	+	+	+	+	+
Δ*tonB1* Δ*ttpB2* Δ*tonB3* Δ*venB* w/p*tonB3*	+	−	−	−	−	−
Δ*tonB1* Δ*ttpD2* Δ*tonB3* Δ*venB* w/p*tonB3*	+	−	−	−	−	−

a Growth was determined by the presence of halos (+) or lack thereof (−) around each iron source indicated after 18 hr at 37°C. Two microliters of each iron source was spotted on the surface of the plates in the following concentrations: FAC, 500 µg/ml; vibriobactin, 1.0 mg/ml; ferrioxamine, 1.0 mg/ml; enterobactin, 1.0 mg/ml; and aerobactin, 1.0 mg/ml. Wild‐type *V. parahaemolyticus* was streaked on the plate as a source of vibrioferrin, and the halo of growth was monitored around the streak.

b The triple mutant strain Δ*tonB1* Δ*tonB2* Δ*tonB3* contained an additional mutation in the *lacZ* gene (Strain AA‐12 in Table A1). This mutation was necessary for previous studies and has been shown to not affect Fe‐siderophore transport (Alice et al., 2008).

Each of the exogenous siderophores *V. cholerae* vibriobactin, *V. parahaemolyticus* vibrioferrin, *Streptomyces* species ferrioxamine, and *E. coli* enterobactin was able to be brought into the cell by using either the TonB1 or the TonB2 system. Single mutants affecting any of the TonB systems were able to utilize these exogenous siderophores. It is only when a Δ*tonB1* mutation is in conjunction with a TonB2 system mutation (Δ*tonB2,* Δ*ttpB2,* or Δ*ttpD2*) or when a triple mutant, missing components of all three TonB systems, is used that the siderophores cannot be brought into the cell. These results provide additional evidence that both TtpB2 and TtpD2 are necessary components of the TonB2 system and must be functional for *V. vulnificus* to utilize exogenous siderophores.

Quadruple mutants, where vulnibactin production is halted due to the deletion of *venB*, were also unable to utilize exogenous siderophores listed above. Complementation of these quadruple mutants with either *tonB1*, *ttpB2*, or *ttpD2* restored the uptake of all of the above exogenous siderophores (Table 4). These quadruple strains were important to assay because they prove that vulnibactin is unable to steal the iron that is bound by the exogenous siderophore and that the exogenous siderophores are indeed the source of iron in this study. These results again demonstrate the essentiality of TtpB2 and TtpD2 in the TonB2 system.

In contrast, it has been shown that aerobactin, a commonly produced siderophore by enterobacteria, can only be brought in through the TonB2 system (Kustusch et al., 2012; de Lorenzo & Martinez, 1988). Consistent with these findings, double and triple mutants behaved as would be anticipated in a similar fashion to single mutants in the TonB2 system preventing aerobactin from being utilized (Table 4).

### 3.4 Complementation of a *V. vulnificus* Δ*ttpB2* must come from *V. vulnificus,* while a Δ*ttpD2* mutant can be complemented by different members of the *Vibrionaceae*

It has previously been demonstrated that the TtpC2 protein from different *Vibrio* species can be substituted for a Δ*ttpC2* mutation in *V. vulnificus* to restore function of the TonB2 system (Kustusch et al., 2012). To better examine the importance and conservation of function of both TtpB2 and TtpD2, proteins from other *Vibrio* species were substituted into a *V. vulnificus* triple mutant (Δ*tonB1* Δ*ttpB2* Δ*tonB3* or Δ*tonB1* Δ*ttpD2* Δ*tonB3*). Complementing plasmids were moved into appropriate strains, and growth assays were performed.

**Figure 5:**
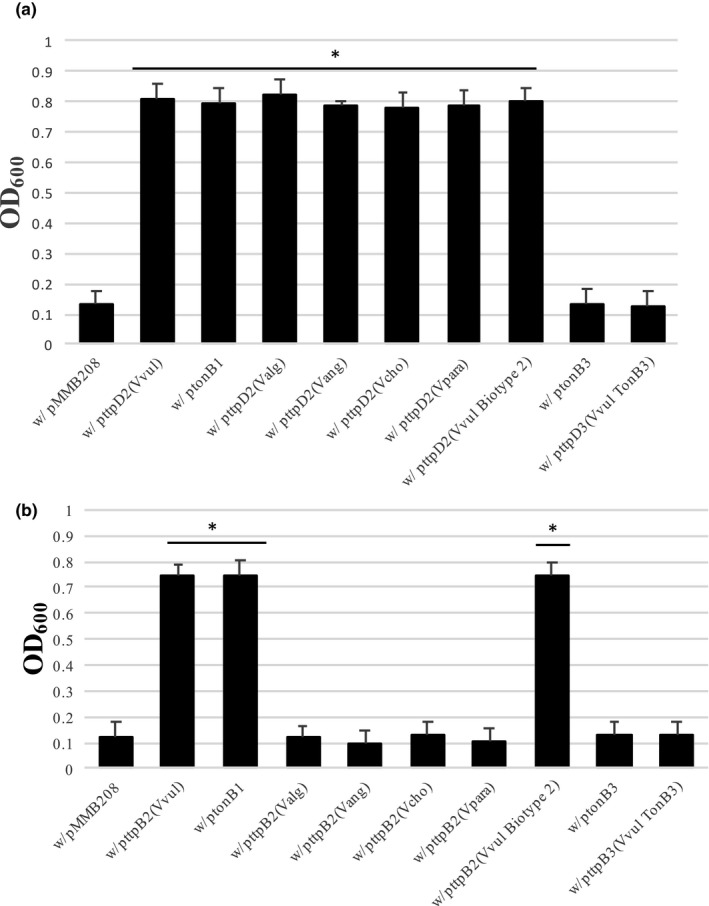
TtpD2, and not TtpB2, can be complemented by other *Vibrio* species to restore function of the TonB2 system. Triple mutants, Δ*tonB1* Δ*ttpD2* Δ*tonB3* (a) or Δ*tonB1* Δ*ttpB2* Δ*tonB3* (b), were used with various complementing plasmids. Overnight cultures were diluted to an optical density at 600 nm (600_nm_) of 0.02 into CM9 media plus 75 µM 2,2′‐dipyridyl (iron‐deplete media), 1 mM IPTG, and 10 µg/ml chloramphenicol. Cultures were grown at 37°C and monitored for growth every 30 min. The average growth at 150 min is depicted for each strain. Strains were grown in triplicate, and significance (*p = <.005) was determined through Student's *t* test

Consistent with the past findings related to Δ*ttpC2*, the Δ*tonB1* Δ*ttpD2* Δ*tonB3* mutant growth was complemented using the majority of other *Vibrio* species' homologous genes (Figure 5a). All TtpD2 proteins from the six *Vibrio* species tested were able to restore growth. Growth in these conditions was not restored to the strains with nonfunctional TonB2 systems, when TtpD3 from the TonB3 system was used for complementation, demonstrating a distinction in roles for TtpD2 and TtpD3.

In contrast to these TtpD2 findings and previous findings with TtpC2, growth was only restored in a TtpB2 mutant when a complementing plasmid restoring TonB1 or a TtpB2 protein from a *V. vulnificus* species (wild‐type or a Biotype 2 strain) was utilized. Results using the Δ*tonB1* Δ*ttpB2* Δ*tonB3* strains can be seen in Figure 5b. Provision of any other TtpB2 protein from different *Vibrio* species or TonB3 was not able to restore growth. These results demonstrate that the TonB2 system has a specificity for its own TtpB2 protein but not TtpD2.

In addition to determining if growth could be restored through complementation with homologous copies of TtpB2 and TtpD2, bioassays were used to examine restoration of the ability to utilize siderophores. As would be expected, bioassays utilizing homologous TtpB2 from other *Vibrio* strains show parallel results as growth assays—homologous strains did not restore siderophore utilization (Table 5). Confirming the ability of the TonB2 system to utilize homologous TtpD2 from other Vibrio species, the TptD2 complemented strains saw restoration of their ability to uptake each siderophore tested (Table 6). These data, along with the data from the growth assays, clearly demonstrate the specific need of a native form of TtpB2, but not TtpD2, for growth and siderophore uptake.

**Table 5:** Complementation of *ttpB2* from other non‐*Vibrio vulnificus* species does not restore the TonB2 system‐mediated uptake of various iron sources^a^

Iron source^b^	TtpB2 from other *Vibrio* species^a^						
	pMMB208	Vvul	Vvul Biotype 2	Vcho	Vpara	Valg	Vang
FAC	+	+	+	+	+	+	+
Vulnibactin	−	+	+	−	−	−	−
Hydroxamate	−	+	+	−	−	−	−
Vibriobactin	−	+	+	−	−	−	−
Vibrioferrin	−	+	+	−	−	−	−
Aerobactin	−	+	+	−	−	−	−
Enterobactin	−	+	+	−	−	−	−
Ferrioxamine	−	+	+	−	−	−	−

a The embedded strain, VSRK615 (Δ*tonB1* Δ*ttpB2* Δ*tonB3* Δ*venB*), contained the complementing plasmid pMMB208 expressing TtpB2 from the various *Vibrio* species listed at the top of the table.

b Growth was determined by the presence of halos (+) or lack thereof (−) around each iron source indicated after 18 hr at 37°C. Two microliters of each iron source was spotted on the surface of the plates in the following concentrations: FAC, 500 µg/ml; vibriobactin, 1.0 mg/ml; ferrioxamine, 1.0 mg/ml; enterobactin, 1.0 mg/ml; and aerobactin, 1.0 mg/ml. Wild‐type *V. vulnificus* and *V. parahaemolyticus* were streaked on the plate as a source of vulnibactin and vibrioferrin, respectively, and the halo of growth was monitored around the streak. A *V. vulnificus* Δ*venB* mutant was streaked onto the plate to test for growth around the hydroxamate siderophore.

**Table 6:** Complementation of *ttpD2* from other *Vibrio* species does restore the TonB2 system‐mediated uptake of various iron sources^a^

Iron source^a^	TtpD2 from other *Vibrio* species^b^						
	pMMB208	Vvul	Vvul Biotype 2	Vcho	Vpara	Valg	Vang
FAC	+	+	+	+	+	+	+
Vulnibactin	−	+	+	+	+	+	+
Hydroxamate	−	+	+	+	+	+	+
Vibriobactin	−	+	+	+	+	+	+
Vibrioferrin	−	+	+	+	+	+	+
Aerobactin	−	+	+	+	+	+	+
Enterobactin	−	+	+	+	+	+	+
Ferrioxamine	−	+	+	+	+	+	+

a The embedded strain VSRK643 (Δ*tonB1* Δ*ttpD2* Δ*tonB3* Δ*venB*) contained the complementing plasmid pMMB208 expressing TtpD2 from the various *Vibrio* species listed at the top of the table.

b Growth was determined by the presence of halos (+) or lack thereof (−) around each iron source indicated after 18 hr at 37°C. Two microliters of each iron source was spotted on the surface of the plates in the following concentrations: FAC, 500 µg/ml; vibriobactin, 1.0 mg/ml; ferrioxamine, 1.0 mg/ml; enterobactin, 1.0 mg/ml; and aerobactin, 1.0 mg/ml. Wild‐type *V. vulnificus* and *V. parahaemolyticus* were streaked on the plate as a source of vulnibactin and vibrioferrin, respectively, and the halo of growth was monitored around the streak. A *V. vulnificus* Δ*venB* mutant was streaked onto the plate to test for growth around the hydroxamate siderophore.

## 4 DISCUSSION

*Vibrio vulnificus* is a gram‐negative opportunistic pathogen that can infect humans as well as eels (Gulig et al., 2005; Hor, Chang, Chang, Lei, & Ou, 2000). In humans, fatal septicemia is often associated with large amounts of iron found in a person's blood due to several chronic or acute conditions (Wright & Morris, 1991). This high concentration of iron is a major risk factor for many bacterial infections, including by *V. vulnificus* (Crosa et al., 2004). Siderophores are small molecular chelators of iron that many microbes secrete in order to sequester iron from their environment (Neilands, 1995). *V. vulnificus* produces two such siderophores, the dominate siderophore, vulnibactin, and a hydroxamate‐type compound (Okujo et al., 1994; Simpson & Oliver, 1983). Once bound to iron, these siderophores first interact with an OM receptor before being internalized. Energy, produced through the PMF in the IM, must be transduced to the OM to allow iron‐bound siderophores into the periplasm, and consequently into the cytosol of the cell. The TonB systems are responsible for harnessing this energy and transferring it to the OM receptor (Braun, 1995; Crosa et al., 2004; Postle & Larsen, 2007). Unlike the well‐studied, single TonB system of *E. coli*, *V. vulnificus* contains three TonB systems (Kustusch et al., 2011).

Previous in silico work identified the potential genes in *V. vulnificus*' three TonB systems. While the TonB1 system has the same three genes (*tonB1, exbB1*, and *exbD1*) as *E. coli*, the TonB2 and TonB3 systems of *V. vulnificus* appeared to have an additional gene, *ttpC*, as well as two ORFs found at the beginning and end of each operon (Kustusch et al., 2012). In this study, we have demonstrated that these two ORFs, now renamed *ttpB2* and *ttpD2*, are essential and necessary for the TonB2 system. We have identified a potential Fur binding site and promoter region upstream of *ttpB2* consistent with previous studies that have alluded to the presence of a Fur binding site through gel shift analysis using a His‐tagged Fur protein (Alice et al., 2008). In addition, our semi‐quantitative RT‐PCR analysis has shown the same, if not greater, up‐regulation of *ttpB2* and *ttpD2* in iron‐deplete versus iron‐rich conditions as compared to *tonB2*. This up‐regulation has also been demonstrated through microarray analysis yielding similar fold expression as well as promoter fusion assays (Alice et al., 2008).

While present in the TonB3 system, we have shown that *ttpB3* and *ttpD3* cannot substitute for their apparent homologs in the TonB2 system. While the orientation and arrangement of the *tonB2* and *tonB3* operons appear similar, they in fact are very different from each other. Every member of the *Vibrionaceae* contains the TonB1 and TonB2 systems, while only a limited few contain the TonB3 system (Kustusch et al., 2011). Overall, the TonB2 and TonB3 systems are only about 35% similar on the amino acid level, with TtpB2‐B3 and TtpD2‐D3 having 30% and 23% homology, respectively. While multiple studies have shown that both the TonB1 and TonB2 systems transport iron‐bound siderophores, other studies have shown that the TonB3 system does not appear to transport any iron‐related product under iron‐limiting conditions (Alice et al., 2008; Kuehl & Crosa, 2009; Kustusch et al., 2012). It appears that the TonB3 system is only transcriptionally activated when grown in human serum or grown in minimal media with glycerol as a sole carbon source with low concentrations of casamino acids (Alice & Crosa, 2012; Alice et al., 2008).

In this study, the essentiality of both TtpB2 and TtpD2 for iron transport was evaluated using growth assays, bioassays, and CAS assays. Our results clearly demonstrate that both proteins are necessary components of the TonB2 system and are required for that system to function. This study has also demonstrated that either the TonB1 or the TonB2 system, with all six proteins present, must be functional for the iron uptake necessary for robust growth in iron‐limited conditions. In contrast, these systems do not need to be present for growth in iron‐rich conditions where freely available iron is readily available. In addition, our CAS assays and bioassays have shown that TtpB2 and TtpB2 are essential for the uptake of both endogenous and exogenous siderophores through the TonB2 system. While most exogenous siderophores could be brought in through the TonB1 or TonB2 system, aerobactin is only brought in by the TonB2 system. Finally, this study has shown that TtpB3 and TtpD3 cannot complement the TonB2 system deletions for growth, in both their natively expressed levels or when artificially overexpressed, in the iron‐limited conditions tested in this study or for the uptake of siderophores.

TtpB2 and TtpD2 are predicted to be 255 and 425 amino acids, respectively. TtpB2 contains a leucine zipper motif (Leu‐X6‐Leu‐X6‐Leu‐X6‐Leu) toward the N‐terminus, while TtpD2 contains a number of TPR motifs. Both of these conserved motifs suggest that TtpB2 and TtpD2 function as part of multiprotein complexes or through protein–protein interactions (Hirst et al., 1996; Schultz et al., 1998). Current work is underway to examine these motifs and the way in which these proteins interact within the TonB2 system.

Our RT‐PCR analysis shows a ~2.5‐fold increase in the expression of *ttpB2* and an 8‐fold increase in the expression of *ttpD2* confirming an up‐regulation of these genes alongside *tonB2* in iron‐depleted conditions relative to iron‐rich.

Previously, both TonB2 and TtpC2 have been shown to be interchangeable among *Vibrio* species to create a functional TonB2 system (Kustusch et al., 2012; Stork et al., 2007)*.* Similarly to other genes in the TonB2 system examined thus far, we demonstrate that *V. vulnificus* TtpD2 function can be complemented with homologs from other *Vibrio* species despite only around 35% amino acid conservation. Surprisingly however, *V. vulnificus* TtpB2, despite having a higher level of conservation (around 50%), cannot be complemented with the homolog from other species. Only the Biotype 2 strain with 97% homology to our wild‐type background was able to complement our *ΔttpB2* mutation. Studies are underway to determine why this protein is unique in that other homologs of TtpB2 cannot complement its function within the *V. vulnificus*' TonB2 system. Studies are also underway to examine which portions of TtpD2, with its low level of conservation, are able to provide the conserved function that exists between species.

Previous studies have shown the importance of *V. vulnificus*' TonB systems and its contribution to growth and virulence (Alice et al., 2008; Kustusch et al., 2012). Here, we demonstrate that two additional proteins, TtpB2 and TtpD2, are essential elements for a functional TonB2 system. In conclusion, TtpB2 and TtpD2 are required for *V. vulnificus* growth and the uptake of endogenous and exogenous siderophores in iron‐limited conditions similar to those the organism experiences within a host. Both the specificity of TtpB2 and the ubiquitous nature of TtpD2 will be further analyzed in future studies.

## CONFLICT OF INTEREST

The authors have no conflicts of interest to disclose.

## AUTHOR CONTRIBUTIONS

R.J.K involved in conceptualization, funding acquisition, and project administration; provided the resources; and wrote the original draft of the article. A.D.B, H.J.P., B.R.Z., E.K.R., J.C.R., M.A.M., E.A.P., M.M.B., and R.J.K. curated the data; involved in formal analysis; investigated the study; designed the methodology; provided the software; supervised, validated, and visualized the study; wrote the review; and edited the manuscript.

## ETHICAL APPROVAL

None required.

## Data Availability

All data are provided in full in the results section of this paper apart from the full protein sequences used to create alignments. These sequences are available at the National Center for Biotechnology Information (NCBI) at http://www.ncbi.nlm.nih.gov/genbank/: *V. vulnificus* CMCP6 (TtpB2 accession number WP_011081323, TtpD2 accession number AAO07317, TtpB3 accession number AAO09346, TtpD3 accession number WP_011078917), *V. vulnificus* biotype 2 ATCC 33149 (TtpB2 accession number WP_039446789, TtpD2 accession number WP_039446781), *V. cholerae* 01 Biovar El Tor (TtpB2 accession number NP_231188, TtpD2 accession number NP_231183), *V. parahaemolyticus* RIMD (TtpB2 accession number NP_799661, TtpD2 accession number NP_799666), *V. alginolyticus* 12G01 (TtpB2 accession number EAS74533, TtpD2 accession number EAS74528), and *V. anguillarum* 775 (TtpB2 accession number AEH33201, TtpD2 accession number AEH33206).

## APPENDIX A

**Table A1:** Bacterial strains and plasmids used in this study

Strain or plasmid	Genotype or relevant characteristic	Reference or source
*V. vulnificus*		
CMCP6	Wild type	J. Rhee
AA‐14	Δ*venB*	Alice et al. (2008)
AA‐6	Δ*tonB1*	Alice et al. (2008)
AA‐7	Δ*tonB2*	Alice et al. (2008)
VSRK600	Δ*ttpB2*	This study
VSRK659	Δ*ttpD2*	This study
AA‐8	Δ*tonB3*	Alice et al. (2008)
AA‐9	Δ*tonB1* Δ*tonB2*	Alice et al. (2008)
VSRK609	Δ*tonB1* Δ*ttpB2*	This study
VSRK625	Δ*tonB1* Δ*ttpD2*	This study
AA‐10	Δ*tonB1* Δ*tonB3*	Alice et al. (2008)
AA‐11	Δ*tonB2* Δ*tonB3*	Alice et al. (2008)
VSRK605	Δ*ttpB2* Δ*tonB3*	This study
VSRK645	Δ*ttpD2* Δ*tonB3*	This study
AA‐12	Δ*tonB1* Δ*tonB2* Δ*tonB3* Δ*lacZ*	Alice et al. (2008)
VSRK611	Δ*tonB1* Δ*ttpB2* Δ*tonB3*	This study
VSRK647	Δ*tonB1* Δ*ttpD2* Δ*tonB3*	This study
AA‐16	Δ*tonB1* Δ*tonB2* Δ*tonB3* Δ*venB*	Alice et al. (2008)
VSRK615	Δ*tonB1* Δ*ttpB2* Δ*tonB3* Δ*venB*	This study
VSRK643	Δ*tonB1* Δ*ttpD2* Δ*tonB3* Δ*venB*	This study
*V. parahaemolyticus*		
RIMD	Wild type	T. Honda
*E. coli*		
S17‐1λpir	*Λpir* lysogen; *thi pro hsdR hsdM* ^+^ *recA* RP4‐2 Tc:Mu‐Km:Tn7; Tp^r^ Sm^r^	Simon, Priefer, Pühler (1983)
Plasmids		
pPCR2.1	TA cloning vector; Amp^r^, Km^r^	Invitrogen
pDM4	Suicide vector with oriR6K; Cm^r^ *sacB*	Milton et al. (1996)
pRK2013	Helper plasmid; Km^r^	Figurski and Helinski, (1979)
pMMB208	Broad‐host‐range expression vector; Cm^r^ *Ptac*	Morales et al. (1991)
p*tonB1*	*V. vulnificus* CMCP6 *tonB1* cloned into pMMB208	Alice et al. (2008)
p*tonB2*	*V. vulnificus* CMCP6 *tonB2* cloned into pMMB208	Alice et al. (2008)
p*tonB3*	*V. vulnificus* CMCP6 *tonB3* cloned into pMMB208	Alice et al. (2008)
p*ttpB2*(Vvul)	*V. vulnificus* CMCP6 *ttpB2* cloned into pMMB208	This study
p*ttpB2*(Vcho)	*V. cholerae* CA401 *ttpB2* cloned into pMMB208	This study
p*ttpB2*(Vpara)	*V. parahaemolyticus* RIMD *ttpB2* cloned into pMMB208	This study
p*ttpB2*(Valg)	*V. alginolyticus* 12G01 *ttpB2* cloned into pMMB208	This study
p*ttpB2*(Vang)	*V. anguillarum* 775 *ttpB2* cloned into pMMB208	This study
p*ttpD2*(Vvul)	*V. vulnificus* CMCP6 *ttpD2* cloned into pMMB208	This study
p*ttpD2*(Vcho)	*V. cholerae* CA401 *ttpD2* cloned into pMMB208	This study
p*ttpD2*(Vpara)	*V. parahaemolyticus* RIMD *ttpD2* cloned into pMMB208	This study
p*ttpD2*(Valg)	*V. alginolyticus* 12G01 *ttpD2* cloned into pMMB208	This study
p*ttpD2*(Vang)	*V. anguillarum* 775 *ttpD2* cloned into pMMB208	This study
p*ttpB3*(Vvul TonB3)	*V. vulnificus* CMCP6 *ttpB3* from the TonB3 system cloned into pMMB208	This study
p*ttpD3*(Vvul TonB3)	*V. vulnificus* CMCP6 *ttpD3* from the TonB3 system cloned into pMMB208	This study
p*ttpB2*(Vvul Biotype 2)	*V. vulnificus* biotype 2 ATCC 33149 *ttpB2* cloned into pMMB208	This study
p*ttpD2*(Vvul Biotype 2)	*V. vulnificus* biotype 2 ATCC 33149 *ttpD2* cloned into pMMB208	This study

Abbreviations: Amp^r^, ampicillin resistant; Cm^r^, chloramphenicol resistant; Km^r^, kanamycin resistant; Sm^r^, streptomycin resistant; Tp^r^, trimethoprim resistant.

**Table A2:** Primers used in this study

Name	Sequence 5′‐3′^a^
SOE PCR	
TTPB2‐1	CGAACTAGTCGCTGCAAAGCGATCGGCAAG
TTPB2‐2	CGACCCGGGGTAAACAAGTGGTGGTCGCTC
TTPB2‐3	ATACCGCGGCCAGTAAGAGCCCGCCCGCGATGTTGAATATG
TTPB2‐4	CATATTCAACATCGCGGGCGGGCTCTTACTGGCCGCGGTAT
TTPD2‐1	CGAACTAGTCAGCAGCACGCAACTCGGTTC
TTPD2‐2	CGACCCGGGGCCATGGCGTTGCTCGTCAAT
TTPD2‐3	ATAATGCGCTCGCGGATGCCAGCAATGTGCTCGCCGACAAA
TTPD2‐4	TTTGTCGGCGAGCACATTGCTGGCATCCGCGAGCGCATTAT
Complementation	
TTPB2_Vvul_For	CGACTGCAGCAACACGTTGGAGATGGCAAA
TTPB2_Vvul_Rev	CGAGGATCCCTGTTGGTTTTCTGCCTGTTG
TTPB2_Vcho_For	CGAGGATCCCATTTCACCGCGAATAAGACG
TTPB2_Vcho_Rev	CGACCCGGGACGAGGCTATTTGATGCGTTA
TTPB2_Vpara_For	CGACTGCAGAGGTGCGAGTGGAGTAGTGGT
TTPB2_Vpara_Rev	CGAGGATCCGTGCGTTTGCTGCTGACGATT
TTPB2_Valg_For	CGACTGCAGTTGGAGAAGGCCGCAACTACA
TTPB2_Valg_Rev	CGACTGCAGTTGGAGAAGGCCGCAACTACA
TTPB2_Vang_For	CGACTGCAGATACTGTGGTTGTTTGCA
TTPB2_Vang_Rev	CGAGGATCCGTGCTGCTGCTGCTGTTTGTT
TTPD2_Vvul_For	CGAAAGCTTAGCGCAGCCAGCGCGTTTTTT
TTPD2_Vvul_Rev	CGAGGATCCGCGCGTGCTGTTTAGGCTATC
TTPD2_Vcho_For	CGACTGCAGTTTGAGCGTGAGGCGATGCTG
TTPD2_Vcho_Rev	CGAGGATCCCAGGTAGGCTGCAAGTAAGTG
TTPD2_Vpara_For	CGACTGCAGCGATGCAGAGCCAAAGCGTAT
TTPD2_Vpara_Rev	CGAGGATCCCATCACCAATAAATGTGGAGC
TTPD2_Valg_For	CGACTGCAGTTTGAAAGGGAAGCAATCCGT
TTPD2_Valg_Rev	CGAGGATCCGTGGGGCATAATTTAGGTGAT
TTPD2_Vang_For	CGAGGATCCGTGGGGCATAATTTAGGTGAT
TTPD2_Vang_Rev	CGAGGATCCCGTTAAACGTTATGGTACGGA
TTPB3_Vvul_For	CGAAAGCTTTCGCCAAGTGTATGTTGAGGC
TTPB3_Vvul_Rev	CGAGGATCCGCGCTGGAGACCATAAACAGG
TTPD3_Vvul_For	CGACTGCAGAGGTTTGGTTGTTCTTGAGTT
TTPD3_Vvul_Rev	CGAGGATCCGCGATTTGCCTGTTGTGTAGA
Semi‐Quantitative RT‐PCR	
TTPB2_RT_For	CTTGCTGCCGTAATCCAACTC
TTPB2_RT_Rev	CATCAATCAAGACATGCCGAT
TTPD2_RT_For	AACTGCGCGTCGAACTGACGC
TTPD2_RT_Rev	TGGCAACAAGCGCTCGAACTT
TONB2_RT_For	CGGTGGAACACTACGCTGCCT
TONB2_RT_Rev	CCGTGGCGTTAGCGCTGGTCT
16srRNA_RT_For	GACTTCACCCCAGTCATGAAC
16srRNA_RT_Rev	CAGAATGCCACGGTGAATACG
GAPDH_RT_For	TTGATTGGCCAGAATTGGAGTTTG
GAPDH_RT_Rev	TGGTTTCAATCACGTGGCCATTGA

a Underlined portion represents restriction enzyme recognition sites.
